# Innovative machine learning-based prediction of early airway hyperresponsiveness using baseline pulmonary function parameters

**DOI:** 10.3389/fmed.2025.1611683

**Published:** 2025-08-04

**Authors:** Hua Yang, Xingru Zhao, Zhuochang Chen, Lihong Yang, Guihua Zhao, Chenxiao Xu, Jinyi Xu

**Affiliations:** ^1^Department of Cardiopulmonary Function, Henan Provincial People’s Hospital, Zhengzhou University People’s Hospital, Zhengzhou, China; ^2^Department of Respiratory and Critical Care Medicine, Henan Provincial People’s Hospital, Zhengzhou University People’s Hospital, Zhengzhou, China

**Keywords:** airway hyperresponsiveness, pulmonary function parameters, asthma, predictive model, machine learning

## Abstract

**Background:**

The Bronchial Provocation Test (BPT) is the gold standard for diagnosing airway hyperresponsiveness (AHR) in suspected asthma patients but is time-consuming and resource-intensive. This study explores the potential of baseline pulmonary function parameters, particularly small airway indices, in predicting AHR and develops a machine learning-based model to improve screening efficiency and reduce unnecessary BPT referrals.

**Methods:**

This retrospective study analyzed baseline pulmonary function data and BPT results from Henan Provincial People’s Hospital (May to September 2024). Data were randomly split into training (69.8%, *n* = 289) and validation (30.2%, *n* = 125) groups using R software (Version 4.4.1). The Least Absolute Shrinkage and Selection Operator (LASSO) was applied to identify the most predictive variables, and 10-fold cross-validation was used to determine the optimal penalty parameter (*λ* = 0.023) to prevent overfitting. Model fit was evaluated using the Akaike Information Criterion (AIC), and a logistic regression model was constructed along with a nomogram.

**Results:**

The optimal model (Model C, AIC = 310.44) included FEV1/FVC%, MEF75%, PEF%, and MMEF75-25%, which demonstrated superior discriminative capacity in both the training (AUC = 0.790, cut-off = 0.354, 95% CI: 0.724–0.760) and validation cohorts (AUC = 0.756, cut-off = 0.404, 95% CI: 0.600–0.814). In the validation cohort, multidimensional validation through calibration plots showed a slope of 0.883. The Net Reclassification Improvement (NRI) for Model C compared to other models was 0.169 (vs. Model A), 0.144 (vs. Model B), and 0.158 (vs. Model D). The Integrated Discrimination Improvement (IDI) and Decision Curve Analysis (DCA) indicated that Model C provided superior predictive performance and a significantly higher net benefit compared to the extreme curves. For instance, the 10th randomly selected patient in the validation cohort showed an 89.80% probability of AHR diagnosis, with a well-fitting model.

**Conclusion:**

This study identifies MEF75%, MMEF75-25%, FEV1/FVC%, and PEF% as effective predictors of early airway hyperresponsiveness in suspected asthma patients. The machine learning-based predictive model demonstrates strong performance and clinical utility, offering potential as a visual tool for early detection and standardized treatment, thereby reducing the risk of symptom exacerbation, lung function decline, and airway remodeling.

## 1 Introduction

Asthma remains a globally prevalent chronic airway disorder, exhibiting high morbidity, persistent therapeutic challenges, and substantial disease burden across all age demographics, with the global patient population exceeding 330 million (1, 2). Recent epidemiological investigations reveal significant disease heterogeneity in different populations. Specifically, data from the 2019 China Pulmonary Health Study led by Prof. Wang Chen’s research team demonstrated an asthma prevalence rate of 4.2% among adults aged ≥20 years, translating to approximately 45.7 million affected individuals nationwide (3). In contrast, prevalence rates are notably higher in other regions, with 8.4% in the United Kingdom and 12.5% in the United States (4). Complementing these cross-country variations, Burnette et al. further identified demographic patterns in asthma distribution, noting that cases predominantly occur in female (69%), Caucasian (75%), and non-Hispanic (69%) individuals, with most diagnoses made during adulthood (5). Notably, clinical management challenges persist across populations, as evidenced by suboptimal treatment outcomes in 55.1–62.0% of patients. Particularly concerning is the subgroup with severe/uncontrolled asthma (SUA), who demonstrate substantially elevated healthcare expenditures compared to mild asthma cases - a disparity highlighting the urgent need for improved therapeutic strategies (5).

Airway hyperresponsiveness (AHR) is a key pathological feature of asthma, referring to excessive and sustained bronchoconstriction in response to both external and internal stimuli (6–8). The bronchial provocation test is the standard method for assessing AHR, but it is complex to perform and carries certain risks, such as the potential to trigger acute attacks or allergic reactions. Thus, such a test is unsuitable for patients with severe asthma or chronic obstructive pulmonary disease (COPD) (9). Although methacholine challenge testing (MCT) is widely used, its complexity and high costs limit its use in primary care settings (10). Studies in the United States indicate that early accurate diagnosis of AHR remains challenging due to the lack of specific biomarkers. Most of asthma patients are managed by primary care physicians (PCPs), while approximately one-third of these patients do not receive the timely treatment, which can lead to the worsening airway inflammation, airway remodeling, and decreased lung function (11, 12). Therefore, there is an urgent need for a simple and effective predictive method to identify high-risk patients early.

Machine learning (ML) is widely applied in the medical field for disease diagnosis and prognosis prediction. By constructing models, ML can deeply analyze medical data, support clinical decision-making, and classify individual disease risks with high precision in the context of complex diseases, thus aiding in more accurate diagnosis, disease progression prediction, and personalized treatment planning. In this study, a large cohort of patients with suspected asthma was enrolled, with their baseline pulmonary function test data and clinical information systematically collected. Through LASSO regression analysis, 4 clinically accessible and safe indicators were identified, and their application value in the diagnosis of AHR was explored in depth. In the process, LASSO regression achieved precise screening of key pulmonary function parameters by penalizing irrelevant variables, aiming to construct a concise and efficient AHR prediction model. This method can prioritize the retention of variables with clear clinical significance, such as small airway indicators, while effectively reducing the risk of model overfitting, thus ensuring that the constructed model possesses both diagnostic accuracy and operational feasibility in routine clinical practice. Notably, a novel ML-based nomogram model for AHR diagnosis was developed, and internal validation was conducted to assess its diagnostic efficacy. This research has the potential to enhance the accuracy and efficiency of clinical diagnosis, promote early intervention and personalized treatment in asthma, reduce the risks of acute exacerbations, lung function decline, and airway remodeling, and may ultimately contribute to improvements in patient outcomes and quality of life.

## 2 Materials and methods

### 2.1 Study subjects

This study, which was part of routine clinical practice, enrolled consecutive patients attending the outpatient clinic of Henan Provincial People’s Hospital from May to September 2024. The research protocol received ethical approval from the Ethics Committee of Henan Provincial People’s Hospital (Approval No. 2024173) in accordance with the Declaration of Helsinki. Written informed consent was obtained from all participants prior to enrollment through standardized documentation procedures.

#### 2.1.1 Inclusion criteria

(1) Patients with suspected asthma symptoms (e.g., recurrent breathlessness, coughing, chest tightness, wheezing) for a duration of ≥2 months.
(2) All patients underwent routine pulmonary function tests and MCT.
(3) Imaging tests showed no significant abnormalities (such as lung masses, bronchiectasis, pulmonary infections, etc.).

#### 2.1.2 Exclusion criteria

(1) Patients aged <16 years.
(2) Patients with coexisting respiratory diseases including pneumonia, lung cancer, allergic bronchopulmonary aspergillosis, or chronic obstructive pulmonary disease (COPD, defined as post-bronchodilator FEV1/FVC < 0.7).
(3) Patients with heart-related wheezing.
(4) Patients with severe systemic diseases or malignant tumors.
(5) Pregnant or lactating women.

### 2.2 Research methods

#### 2.2.1 Data collection

##### 2.2.1.1 Basic information

Demographic variables were extracted from the hospital’s electronic medical records, including age, sex, body mass index (BMI), symptoms, symptom duration, and medical history.

##### 2.2.1.2 Routine pulmonary function parameters

Pulmonary ventilation function and bronchial provocation tests (BPT) were performed using a Jaeger MasterScreen pulmonary function instrument (CareFusion, Hochberg, Germany) in accordance with the standards established by the American Thoracic Society/European Respiratory Society (ATS/ERS): each patient completed at least 3 technically valid maneuvers, and the best results were recorded (13). The relevant pulmonary function parameters collected included: forced vital capacity as a percentage of predicted value (FVC%pred), forced expiratory volume in 1 s as a percentage of predicted value (FEV1%pred), the ratio of forced expiratory volume in 1 s to forced vital capacity as a percentage of predicted value (FEV1/FVC%pred), peak expiratory flow as a percentage of predicted value (PEF%pred), maximal expiratory flow at 50% of forced vital capacity as a percentage of predicted value (MEF50%pred), maximal expiratory flow at 25% of forced vital capacity as a percentage of predicted value (MEF25%pred), maximal expiratory flow at 75% of forced vital capacity as a percentage of predicted value (MEF75%pred), and maximal mid-expiratory flow between 75 and 25% of forced vital capacity as a percentage of predicted value (MMEF 75-25%pred).

##### 2.2.1.3 Imaging examination

Chest high-resolution computed tomography (HRCT) scans were performed using a SoMATOM Siemens Sensation 64-slice spiral CT scanner. All HRCT images were independently reviewed by two radiologists.

#### 2.2.2 Data cleaning and standardization

##### 2.2.2.1 Data inspection, cleaning and standardization

The raw data obtained were sorted and integrated based on key information from the included patients. To ensure the reliability and accuracy of subsequent analyses, data with incomplete information, duplicates, unclear classifications, or outliers were excluded from the dataset. Additionally, corrections and normalization were performed on the variable names and measurement units to ensure data consistency and comparability.

##### 2.2.2.2 Missing data handling

Variables with a missing rate greater than 30% were excluded from the analysis. For variables with a missing rate ≤ 30%, missing values were imputed using multiple imputation by chained equations (MICE). Subsequent multivariate analyses were conducted using the imputed dataset. (Missing data handling. Variables with a missing rate greater than 30% were excluded from the analysis (none in this study). For variables with a missing rate ≤30%, including pulmonary function parameters (FVC%pred: 8.2%; FEV1%pred: 11.5%; FEV1/FVC%pred: 6.9%; PEF%pred: 13.7%), missing values were imputed using multiple imputation by chained equations (MICE). The MICE procedure included 20 imputed datasets, with predictors incorporating all variables in the analysis (consistent with the final multivariate model). Subsequent multivariate analyses were conducted using pooled results from the imputed datasets).

#### 2.2.3 Statistical methods

Statistical analysis was performed using R software (Version 4.4.1). Categorical data were expressed as frequency (*n*) and percentage (%), while normally distributed continuous data were presented as mean ± standard deviation (SD), and non-normally distributed continuous data were presented as median (M) with interquartile range (Q1, Q3). For comparing continuous data, if the data were normally distributed with homogeneity of variance, an independent-samples t-test was used; otherwise, the Wilcoxon rank-sum test was applied. Categorical data were analyzed using the chi-square test or Fisher’s exact test.

Initially, all selected predictor variables were included in a LASSO regression analysis to identify the most valuable diagnostic predictors. The penalty parameter (*λ*) was selected using 10-fold cross-validation to avoid overfitting, with λ values ranging between λ_min (the λ that minimizes model estimation error) and λ_1se (the λ that maintains model estimation error within an acceptable range). Statistically significant diagnostic predictors were then selected. A nomogram based on the Logistic regression model was constructed using the “rms” package in R, and the receiver operating characteristic (ROC) curve was plotted using the “pROC” package to evaluate the model’s reliability and validity. After model development, its predictive performance was assessed in both the training and validation cohorts. The evaluation included calibration, calculation of net reclassification improvement (NRI), integrated discrimination improvement (IDI), and clinical decision curve analysis (DCA) to comprehensively assess the diagnostic performance of the model. A significance level of *p* < 0.05 was set.

## 3 Results

### 3.1 Patient recruitment and baseline profile

#### 3.1.1 Screening and enrollment process

**Figure 1:**
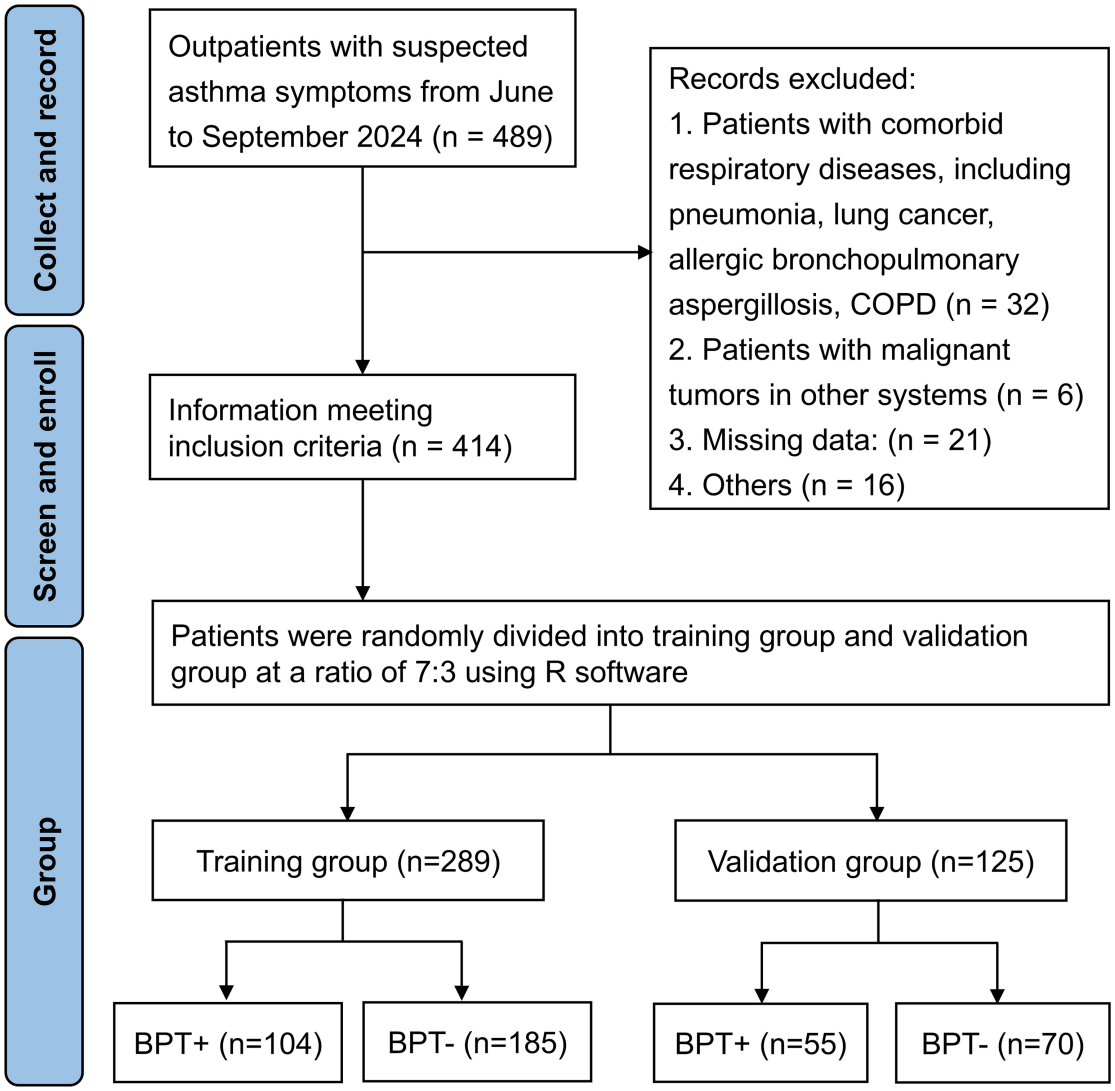
The flow diagram for the screening and enrollment process.

From June to September 2024, a total of 489 outpatients presenting with suspected asthmatic symptoms were initially screened. After rigorous assessment, 414 patients with complete clinical data who met the predefined inclusion criteria were enrolled in the study. The exclusion criteria were applied to 75 patients, with the following breakdown: 32 cases with comorbid respiratory conditions (including pneumonia, lung cancer, allergic bronchopulmonary aspergillosis, and chronic obstructive pulmonary disease [COPD]); 6 cases with malignancies in other systems; 21 cases with incomplete or missing data; and 16 cases excluded due to other unspecified reasons. A flow diagram illustrating this process is provided in Figure 1.

#### 3.1.2 Baseline characteristics

The patients were randomly divided into the training group and validation group at a 7:3 ratio via the sample() function in R software. In the training group, there were 104 patients with positive BPT, with a median age of 52 years (IQR: 32, 61), of which 48 were male (46.2%), a lower proportion than female patients. In the validation group, there were 55 patients with positive BPT, with a median age of 44 years (IQR: 29.5, 57), of which 18 were male (32.7%), also lower than the proportion of female patients. No significant differences were observed in age, sex, BMI, and other baseline characteristics between the training and validation groups (*p* > 0.05). In both the training and validation groups, univariate logistic regression analysis of pulmonary function indices between BPT-negative and BPT-positive groups showed statistically significant differences in 7 variables (*p* < 0.05) (Table 1).

**Table 1:** Basic clinical characteristics of the validation and training groups.

Characteristics	Validation cohort (*n* = 125)			Training cohort (*n* = 289)		
	Negative	Positive	p.overall	Negative	Positive	p.overall
	*N* = 70	*N* = 55		*N* = 185	*N* = 104	
Gender			0.332			0.930
Female	40 (57.1%)	37 (67.3%)		102 (55.1%)	56 (53.8%)	
Male	30 (42.9%)	18 (32.7%)		83 (44.9%)	48 (46.2%)	
Age	46.0 [32.0; 57.0]	44.0 [29.5; 57.0]	0.519	44.0 [33.0; 56.0]	52.0 [32.0; 61.0]	0.069
BMI	24.5 [22.6; 27.0]	25.5 [22.4; 27.3]	0.551	24.7 [22.3; 27.3]	24.7 [22.7; 27.4]	0.720
FEV1/FVC%pred	99.0 [93.0; 104]	88.0 [77.5; 97.0]	<0.001	98.0 [90.0; 103]	84.5 [71.0; 96.0]	<0.001
MEF50%pred	78.4 (25.5)	63.2 (21.4)	<0.001	76.5 [61.0; 94.0]	59.0 [38.5; 77.5]	<0.001
MEF25%pred	58.5 [43.2; 82.8]	38.0 [26.5; 52.0]	<0.001	57.0 [39.0; 77.0]	37.0 [26.0; 49.2]	<0.001
FEV1%pred	98.6 (14.3)	87.5 (14.2)	<0.001	98.0 [86.0; 107]	81.5 [71.8; 97.0]	<0.001
MEF75%pred	93.7 (23.9)	73.5 (21.4)	<0.001	92.5 (23.4)	67.3 (25.2)	<0.001
PEF%pred	99.4 (19.9)	86.7 (18.4)	<0.001	99.8 (19.8)	83.0 (17.9)	<0.001
FVC%pred	106 (14.6)	102 (13.4)	0.151	105 [95.0; 117]	101 [88.0; 112]	0.015
MMEF75-25%pred	70.5 [56.2; 88.2]	51.0 [36.5; 63.5]	<0.001	68.0 [55.0; 85.0]	47.0 [29.8; 58.5]	<0.001

*p* < 0.05 in the table indicates that the differences are statistically significant.

### 3.2 Model development

#### 3.2.1 Dimensionality reduction

**Figure 2:**
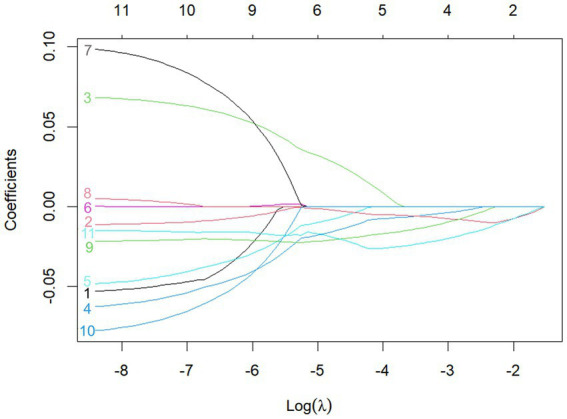
The plot of optimal feature subset selection using LASSO regression.

**Figure 3:**
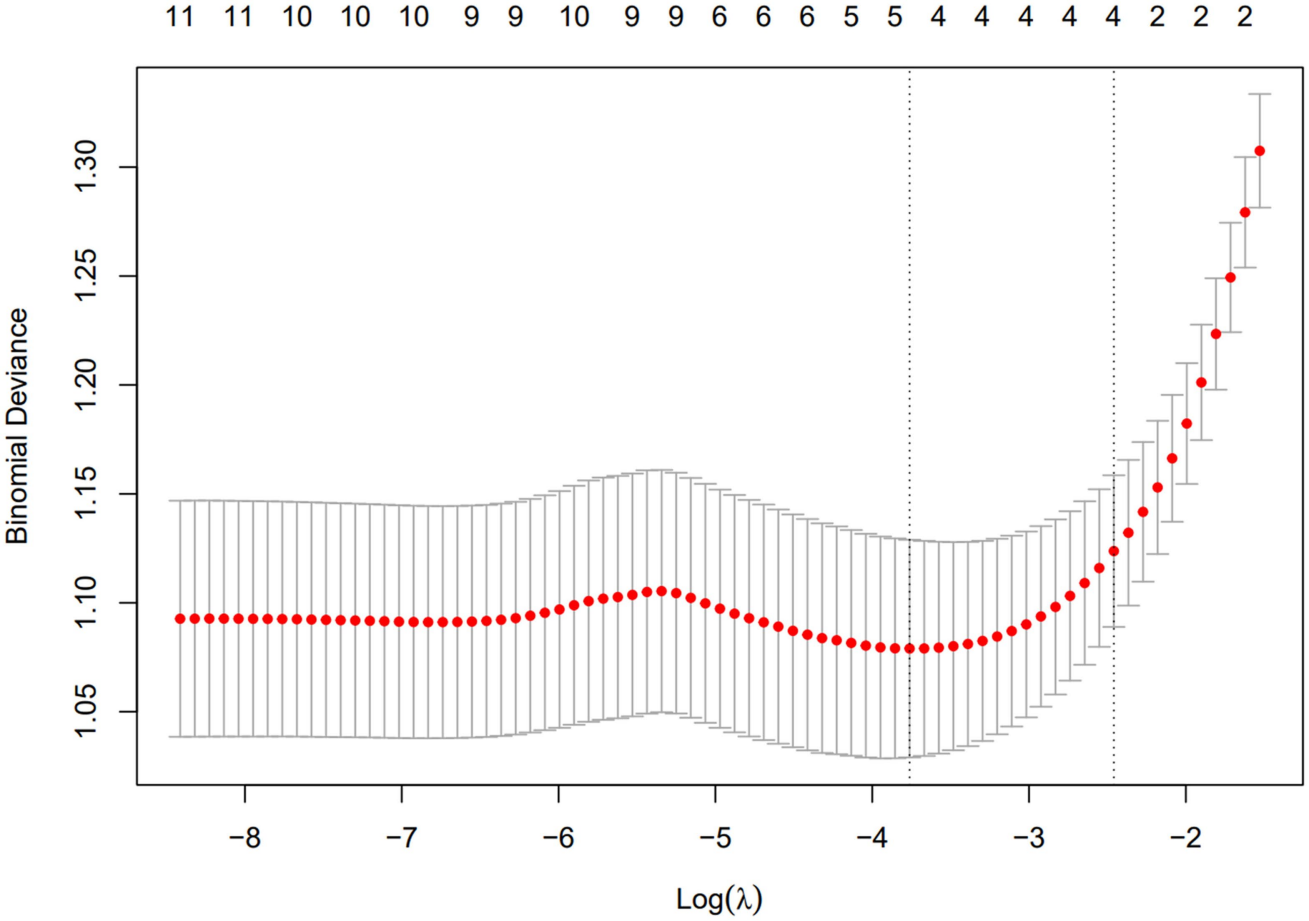
The plot of binomial deviance vs. Log(*λ*) using LASSO regression.

A total of 11 diagnostic indicators were included in this study (Table 1). LASSO regression was used to select features from the demographic characteristics, pulmonary function testing indicators, and other diagnostic-related variables. A 10-fold cross-validation method was applied to select the optimal features corresponding to the tuning parameter *λ*_min_ (the minimum λ criterion), resulting in the best feature subset (Figure 2). The trajectory of the coefficients for each diagnostic predictor was observed as the log of λ changed in the LASSO algorithm (Figure 3). The tuning parameter λ_min_ for LASSO regression was determined to be 0.023 (log(λ_min_) = −3.761) through 10-fold cross-validation. Based on λ_min_, four non-zero coefficient features, including FEV1/FVC%pred, PEF%pred, MEF75%pred and MMEF 75-25%pred, were selected, forming the optimal feature subset.

#### 3.2.2 Development of four predictive models

Based on the results of LASSO regression, four predictive models for AHR diagnosis were constructed using the selected indicators: FEV1/FVC%pred, PEF%pred, MEF75%pred, and MMEF 75-25%pred. The models are as follows: model A (MMEF 75-25%pred); model B (FEV1/FVC%pred, PEF%pred, MEF75%pred); model C (FEV1/FVC%pred, PEF%pred, MEF75%pred, MMEF 75-25%pred); model D (MEF75%pred, MMEF 75-25%pred). The goodness of fit and Akaike Information Criterion (AIC) for each model were calculated, and model C (AIC: 310.44) was selected as the optimal model (Table 2).

**Table 2:** AIC values of the four models.

Model	Variables	Coefficient	95% CL	*p*-value	AIC
Model A	MMEF 75-25%pred	−0.05	(−0.06, −0.04)	<0.001***	312.65
Model B	FEV1/FVC%pred	−0.02	(−0.04, −0.0002)	0.06.	314.19
	MEF75%pred	−0.02	(−0.04, −0.004)	0.02*	
	PEF%pred	−0.01	(−0.04, 0.002)	0.09.	
Model C	FEV1/FVC%pred	−0.01	(−0.03, 0.01)	0.36	310.44
	MEF75%pred	−0.003	(−0.03, 0.02)	0.84	
	PEF%pred	−0.02	(−0.04, −0.001)	0.04*	
	MMEF 75-25%pred	−0.31	(−0.06, -0.006)	0.02*	
Model D	MEF75%pred	−0.02	(−0.04, −0.001)	0.07.	311.23
	MMEF 75-25%pred	−0.03	(−0.06, −0.008)	0.01*	

Signif.codes: 0‘***’0.001 ‘**’0.01 ‘*’0.05 ‘.’0.1‘’1.

#### 3.2.3 Nomogram-based diagnostic prediction model

**Figure 4:**
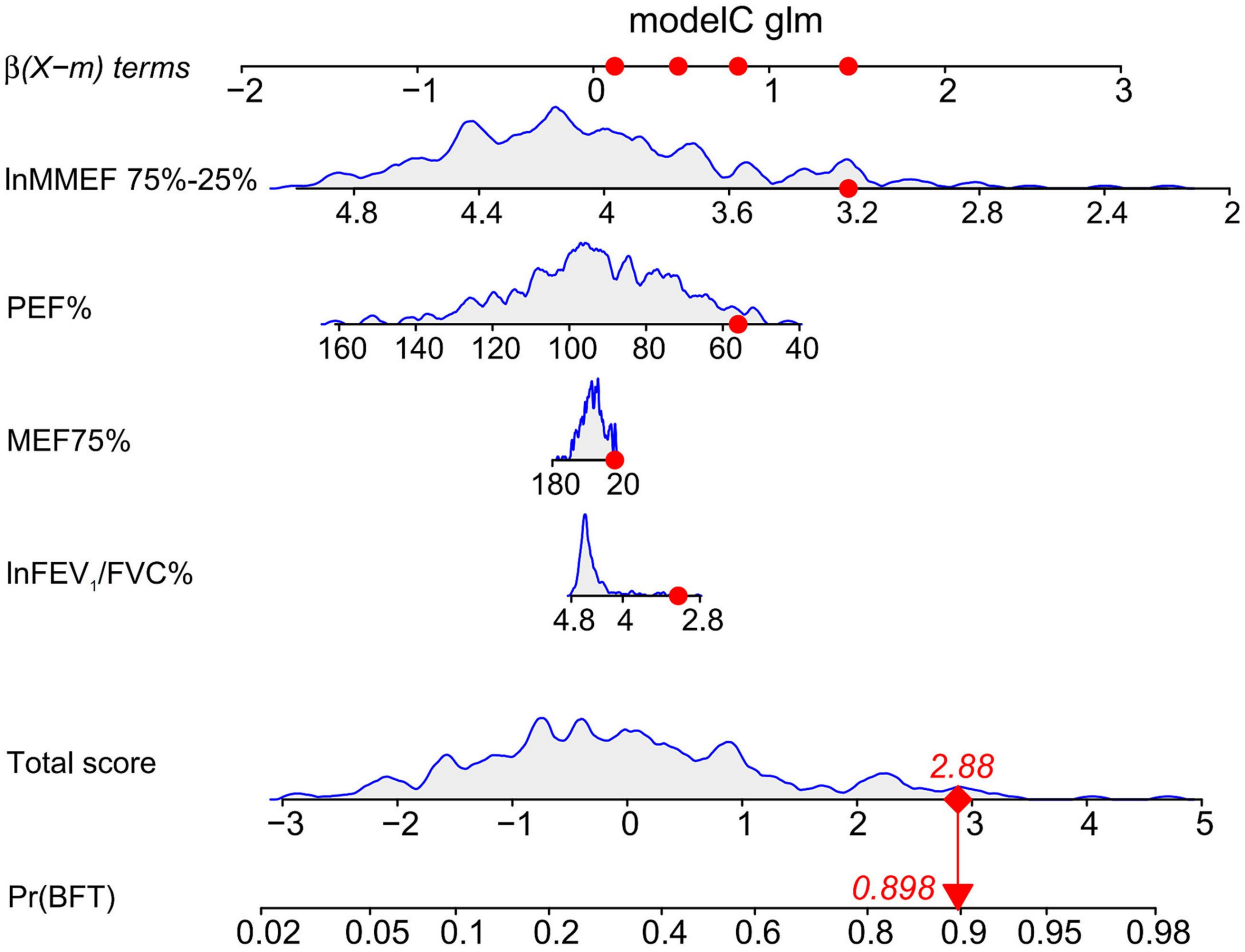
Nomogram of model C using the 10th patient in the study as an example.

A nomogram was constructed based on the selected optimal model, model C (AIC: 310.44), for visualization. Using the 10th patient in the study as an example (Figure 4), each variable’s value in the nomogram corresponds to a specific score. The total score is obtained by summing the individual scores of all variables. The probability of AHR is displayed below the total score, with this patient having a diagnosis probability of 89.80%. The model demonstrates a good fit.

#### 3.2.4 Model performance evaluation

During the model-construction process, the study subjects were divided into two datasets: the training group, which was used to develop the optimal fit model, and the validation group, which was used for internal validation of the model’s predictive performance. After screening the optimal model, its prediction performance was evaluated in both the training and validation groups from four key aspects: calibration, Net Reclassification Index (NRI), Integrated Discrimination Improvement Index (IDI), and Decision Curve Analysis (DCA).

**Figure 5:**
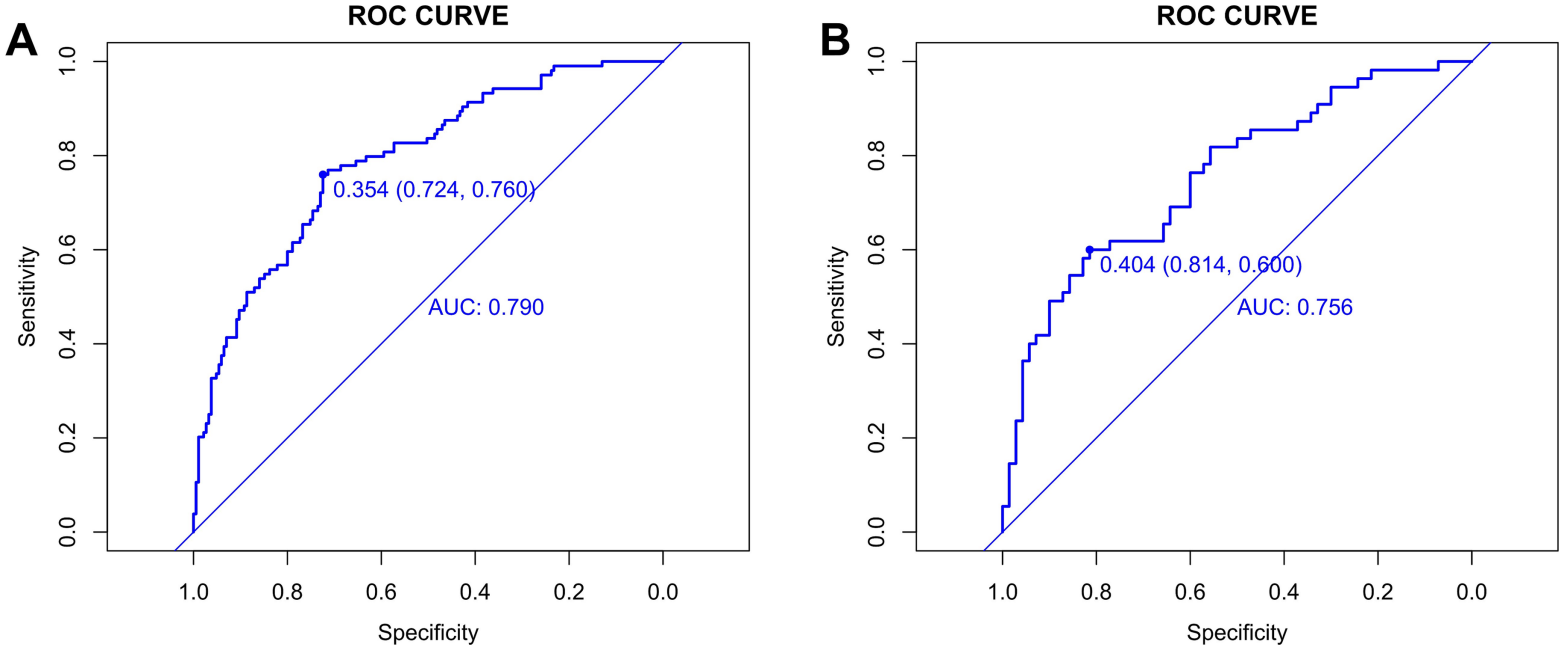
Cut-off values for the training **(A)** and validation **(B)** groups.

Before evaluation, the optimal cut-off values for continuous variables were determined using the surv_cutpoint function from the “survminer” R package. For the training group, the cut-off value was 0.354 (95% CI: 0.724–0.760) (Figure 5A), and for the validation group, it was 0.404 (95% CI: 0.600–0.814) (Figure 5B). The training cohort achieved an AUC of 0.790 with the cut-off value of 0.354, while the validation cohort achieved an AUC of 0.756 with the cut-off value of 0.404. The AUC values ranged from 0.5 to 1, with higher values indicating better model performance. An AUC closer to 1 reflects superior predictive accuracy.

A DeLong test confirmed no statistically significant difference in AUC between cohorts (*p* = 0.509; *z*-statistic = 0.662) (Supplementary Figure S1), indicating the observed performance gap (0.034) falls within random variation. The non-significant *p*-value (>0.05) and *z*-statistic (absolute value <1.96) collectively suggest that the AUC difference stems from random error rather than systematic performance disparities, demonstrating the model’s statistical robustness and consistent performance across cohorts. Together with the model’s moderately high AUC, good calibration, and clinical net benefit, these findings demonstrate that Model C possesses sufficient predictive accuracy and stable performance in new samples, supporting its utility as a reliable tool for early screening of airway hyperresponsiveness (AHR) in suspected asthma patients and reinforcing the clinical relevance of the study conclusions.

##### 3.2.4.1 Calibration evaluation

**Figure 6:**
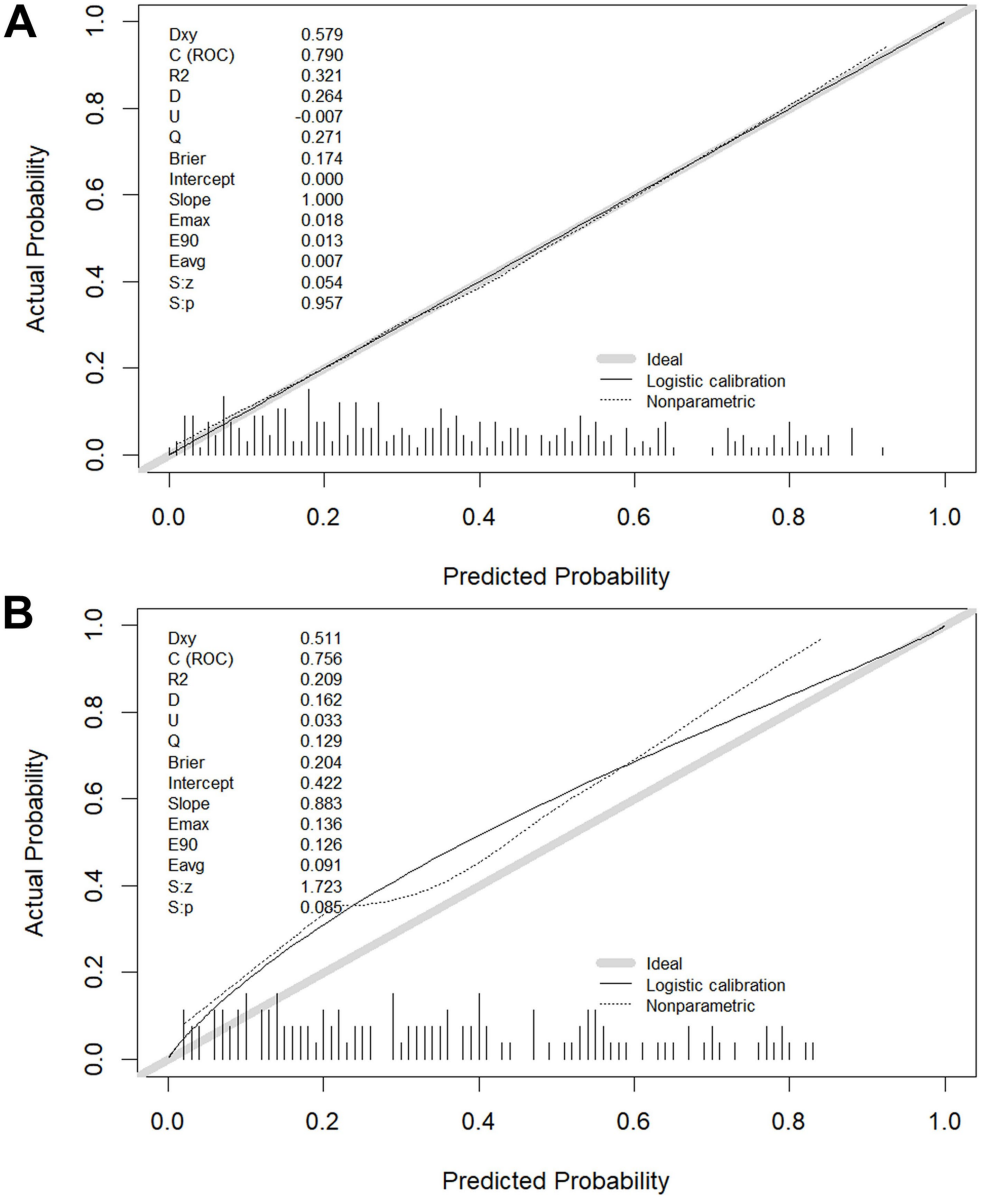
Calibration plots in the training **(A)** and validation **(B)** groups.

The calibration of the model C prediction model in this study was evaluated by plotting calibration curves for both the training (slope = 1.000) (Figure 6A) and validation (slope = 0.883) (Figure 6B) groups.

##### 3.2.4.2 NRI index calculation

**Figure 7:**
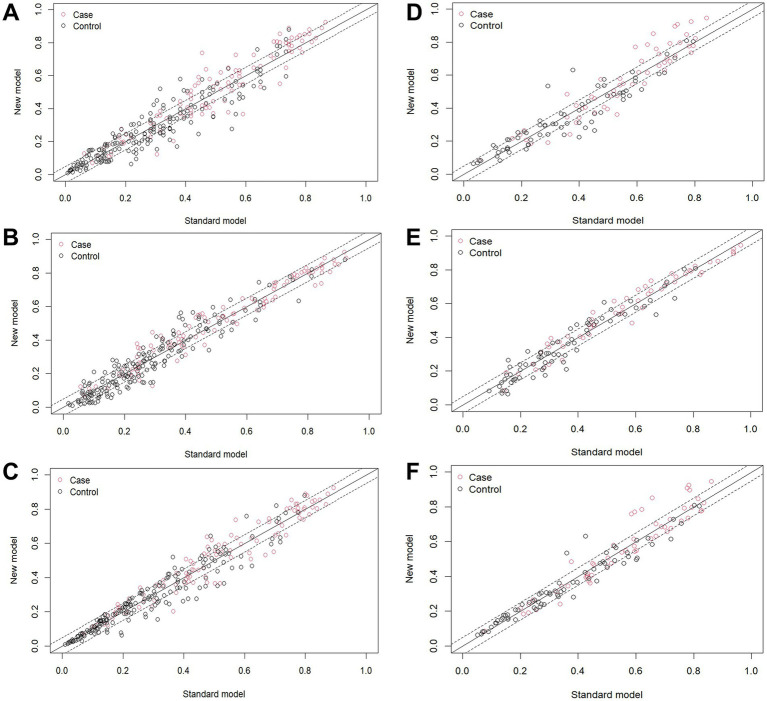
NRI index calculation. In the training group, the NRI value for **(A)** model C vs. model A, **(B)** model C vs. model D, **(C)** model C vs. model B; In the validation group, the NRI value for **(D)** model C vs. model A, **(E)** model C vs. model B, **(F)** model C vs. model D.

In the training group, the results of the comparisons among the three models are as follows: When model C was compared with the other three models, the NRI values were as follows: the NRI value for model C vs. model A was 0.273 (Figure 7A); the NRI value for model C vs. model D was 0.175 (Figure 7B); and the NRI value for model C vs. model B was 0.111 (Figure 7C). These results indicate that model C has superior classification ability, enabling more accurate prediction of AHR. In the validation group, when model C was compared with the other three models, the following NRI values were obtained: the NRI value for model C vs. model A was 0.169 (Figure 7D); the NRI value for model C vs. model B was 0.144 (Figure 7E); and the NRI value for model C vs. model D was 0.158 (Figure 7F). These results suggest that model C exhibits better discriminatory performance, with a clear advantage in predicting AHR.

##### 3.2.4.3 IDI index calculation

Training group: When model C is the new model and model B is the old model, the IDI value is 0.0115 [95% CI: −0.0021 – 0.0252], with a *p*-value of 0.0976; when model C is the new model and model A is the old model, the IDI value is 0.0269 [95% CI: 0.0086–0.0452], with a *p*-value of 0.00403; when model C is the new model and model D is the old model, the IDI value is 0.0141 [95% CI: −0.0003 – 0.0285], with a *p*-value of 0.0555.

Validation group: When model C is the new model and model B is the old model, the IDI value is 0.0115 [95% CI: −0.0021 – 0.0252], with a *p*-value of 0.0976; when model C is the new model and model A is the old model, the IDI value is 0.0269 [95% CI: 0.0086–0.0452], with a *p*-value of 0.0040; when model C is the new model and model D is the old model, the IDI value is 0.0128 [95% CI: 0.0015–0.024], with a *p*-value of 0.0258.

##### 3.2.4.4 DCA

**Figure 8:**
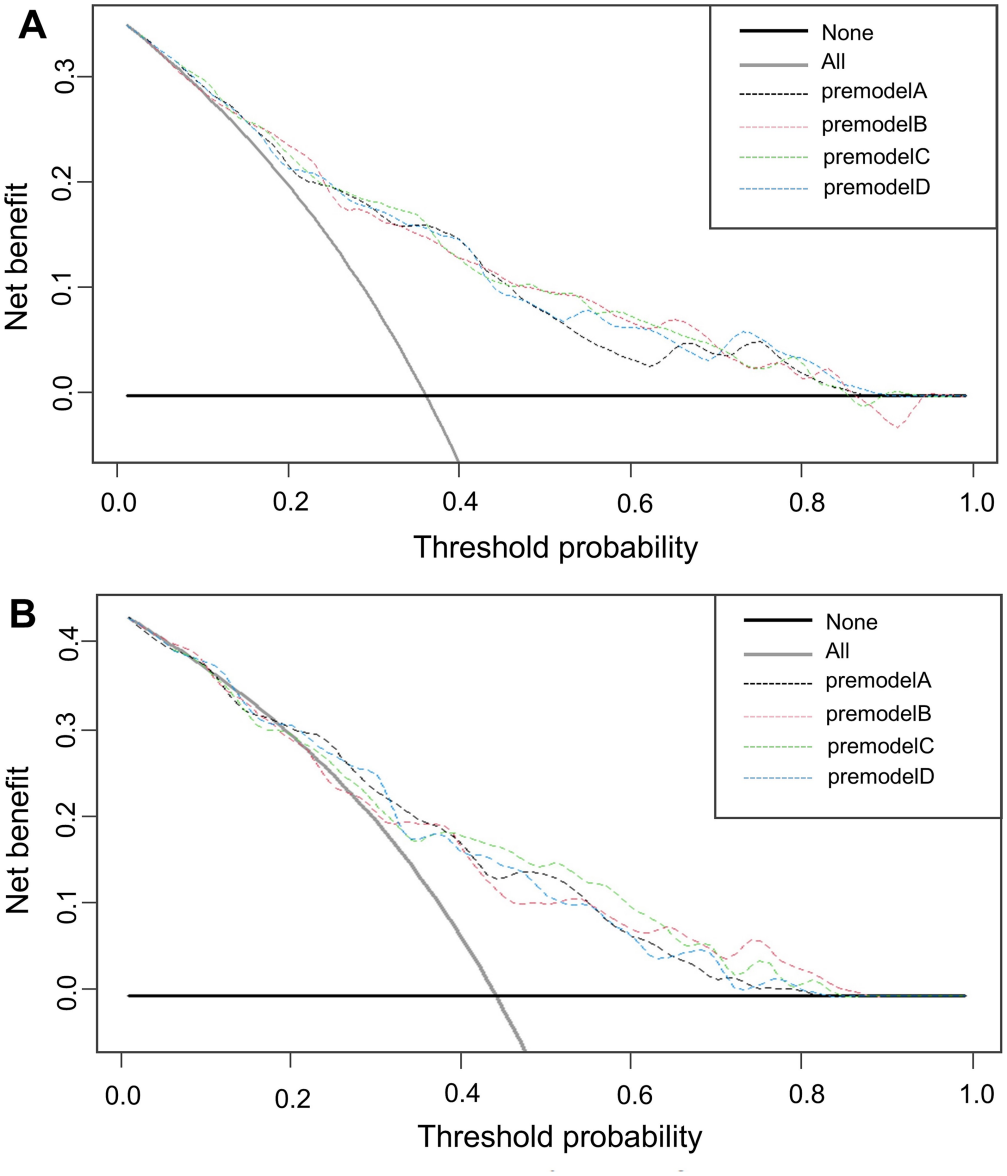
DCA curves for the training **(A)** and validation **(B)** groups.

The x-axis of the graph represents the threshold probability, while the y-axis indicates the net benefit, calculated as the benefit minus the harm. From Figure 8, it is evident that in both the training (Figure 8A) and validation (Figure 8B) groups, model C demonstrates a significantly higher net benefit compared to the extreme curves, indicating its superior performance.

## 4 Discussion

This study developed and validated four models to predict airway hyperresponsiveness (AHR) in suspected asthma patients, with model C showing the best predictive performance. We identified FEV1/FVC%, PEF%, MEF75%, and MMEF75-25% as the optimal parameters for predicting AHR. This is the first study to apply machine learning (ML) algorithms combining small airway function indices, FEV1, and peak expiratory flow (PEF) for AHR prediction. Although previous studies have explored the role of individual indicators (14–16), the innovation of this study lies in its early-stage diagnostic approach, which integrates baseline pulmonary function parameters to exclude the possibility of asthma, providing a simple method for identifying patients requiring referral to MCT, thereby avoiding unnecessary tests. It also explores the potential application of baseline lung function variables in the diagnosis of AHR in suspected adult asthma patients.

The results of this study indicate that AIC balances the model’s complexity with data fit. A lower AIC value indicates a better model fit. Ultimately, model C (AIC: 310.44) was selected as the optimal model and visualized through a nomogram. After model development, we assessed its predictive performance in both the training and validation groups. The validation cohort achieved an AUC of 0.756 with the cut-off value of 0.404. The AUC values ranged from 0.5 to 1, with higher values indicating better model performance. An AUC closer to 1 reflects superior predictive accuracy. Calibration evaluation showed consistency between the predicted and actual risk, with model C’s calibration curve approaching a straight line with a slope near 1, indicating good concordance between predicted and observed probabilities, thus demonstrating high prediction accuracy. When NRI is greater than 0, it indicates that the new model outperforms the old model in classification ability, accurately reclassifying individuals into the correct risk categories. A larger IDI indicates stronger predictive capability for the new model, and when IDI > 0, it signifies significant improvement in predictive power compared to the old model. The DCA graph shows two dashed lines representing net benefits for no treatment and universal treatment, with other curves compared to these lines. The analysis of these results shows that model C demonstrates higher net benefit and stronger predictive value in both groups.

Small airway dysfunction is a key pathological feature of asthma, and early-stage asthma patients may experience inflammation and narrowing in the small airways, increasing airflow resistance (17). In routine pulmonary function tests, a reduction in any two of FEF50%, FEF75%, or FEF25-75% below 65% suggests small airway dysfunction (18–20). While small airways (diameter < 2 mm) contribute minimally to airflow resistance under normal conditions, dysfunction in these airways significantly increases airway resistance and is closely related to AHR, asthma severity, and control level (21–24). Studies show that small airway dysfunction increases the risk of AHR, and the combination of FENO, FEF50%, and FEF25-75% effectively predicts AHR in patients with normal FEV1 (25). Chinese experts suggest that the small airways are the primary site of airway inflammation and remodeling in asthma patients, particularly in preschool children, where small airway dysfunction is associated with AHR and severe airflow obstruction (26). FEF25-75%, a key indicator in routine pulmonary function tests, predicts AHR in patients with respiratory symptoms and has significant value in early asthma exacerbations and bronchial hyperresponsiveness (14, 15). French research first demonstrated that small airway obstruction, assessed by FEF25-75%, can lead to persistent AHR and increased risk of adverse outcomes (16), with changes in FEF25-75% correlating with the severity of newly diagnosed asthma and AHR (27, 28). Israeli studies highlight that baseline FEF50% can effectively exclude AHR and reduce misdiagnosis risk (29). Studies also indicate that minimum PEF is closely associated with AHR in asthma patients, and adjusted Min%Max PEF correlates well with airway responsiveness in inhalation provocation tests. Real-time PEF monitoring has potential in predicting and detecting acute exacerbations in severe asthma patients, and PEF trajectory-derived predictors can effectively monitor disease deterioration, serving as a convenient alternative indicator for AHR (30, 31). Additionally, some studies show that children with asthma typically demonstrate a decrease in PEF 1.34 days before symptom onset, and early PEF monitoring aids in preventing acute exacerbations by enhancing treatment (32). However, Dutch experts believe that PEF variability can serve as a diagnostic tool for AHR, but single indicators cannot completely replace MCT (33). Literature reports indicate that the FEV1/FVC ratio in children with persistent asthma is lower than in healthy children, with similar trends observed in obese asthma patients (34). Moreover, research by Brazilian experts such as Mingotti suggests that an FEV1/FVC ratio near the lower limit of normal indicates poor clinical prognosis in asthma patients without airway obstruction (35). Based on these findings, the model C in this study incorporates indicators such as FEV1/FVC%, PEF%, MEF75%, and MMEF75-25%, which are considered the optimal parameters for predicting AHR, providing important references for the clinical diagnosis and disease management of asthma patients.

In conclusion, we constructed an accurate model using real-world data that can diagnose airway hyperresponsiveness in asthma patients based on baseline lung volume measurement indices. This machine learning-based model demonstrates outstanding performance in predicting AHR, with the potential to enhance clinical asthma diagnosis.

Limitations of this study include its retrospective design, with clinical data sourced from outpatient records and testing systems. Relevant variables such as smoking history and allergy history were not included in the analysis due to missing data, which may introduce bias into the predictions. Future prospective studies should prioritize the systematic collection of smoking-related data (including smoking duration, intensity, and cessation status) and allergy history, aiming to clarify their roles in predicting AHR and further optimize the model framework. Furthermore, this study was conducted at a single center in Henan Provincial People’s Hospital, and the geographic limitations of the patient population—compounded by regional differences in environmental exposures (e.g., biomass fuel use), allergen distributions, and genetic factors—may restrict the external generalizability and applicability of the results. The confinement to a single seasonal window (May–September 2024) in Henan, during which elevated pollen levels and viral infections might have influenced airway hyperresponsiveness prevalence, adds another layer of contextual limitation. Additionally, the underrepresentation of elderly patients and the gender imbalance in the sample (with a higher proportion of female participants) could affect the model’s performance across diverse demographic subgroups. Importantly, the current model is specifically developed and validated for patients with suspected asthma, and its applicability to other respiratory diseases (e.g., COPD or interstitial lung disease) has not been evaluated. These conditions exhibit distinct pathophysiological features—such as irreversible airflow obstruction in COPD or diffuse parenchymal damage in interstitial lung disease—that may alter pulmonary function parameters beyond the scope of the model’s design, which is rooted in asthma-specific characteristics. Similarly, the model’s validity in larger elderly cohorts requires dedicated assessment, given the limited representation of this population in the current dataset. While external validation efforts involving geographically and demographically distinct centers (Beijing, Guangzhou, Sichuan) are underway, future large-scale, multicenter clinical studies spanning multiple seasons should incorporate subgroup analyses by region and demographics to assess model robustness, thereby enhancing population representativeness and result stability.

## 5 Conclusion

The results of the multifactorial analyses in this study indicate that MEF75%, MMEF75-25%, FEV1/FVC%, and PEF% are effective indicators for predicting early airway hyperresponsiveness in suspected asthma patients. The diagnostic prediction model developed using machine learning methods demonstrated good predictive performance and clinical applicability in internal validation. It holds potential as a visual tool to aid in the early identification of mild asthma patients, ensuring timely diagnosis and standardized treatment, thereby reducing the risks of acute symptom exacerbation, pulmonary function decline, and airway remodeling.

## Data Availability

The original contributions presented in the study are included in the article/Supplementary material, further inquiries can be directed to the corresponding authors.

## References

[1] Dubin S Patak P Jung D. Update on asthma management guidelines. Mo Med. (2024) 121:364–7. Available online at: https://pmc.ncbi.nlm.nih.gov/articles/PMC11482852/ PMID: 39421468 PMC11482852

[2] Huang K Wang W Wang Y Li Y Feng X Shen H. Evaluation of a global initiative for asthma education and implementation program to improve asthma CARE quality (CARE4ALL): protocol for a multicenter, single-arm study. JMIR Res Protoc. (2025) 14:e65197. doi: 10.2196/65197, PMID: 39778197 PMC11754978

[3] Huang K Yang T Xu J Yang L Zhao J Zhang X. Prevalence, risk factors, and management of asthma in China: a national cross-sectional study. Lancet. (2019) 394:407–18. doi: 10.1016/S0140-6736(19)31147-X, PMID: 31230828

[4] Suruki RY Daugherty JB Boudiaf N Albers FC. The frequency of asthma exacerbations and healthcare utilization in patients with asthma from the UK and USA. BMC Pulm Med. (2017) 17:74. doi: 10.1186/s12890-017-0409-3, PMID: 28449686 PMC5406966

[5] Lugogo N Judson E Haight E Trudo F Chipps BE Trevor J. Severe asthma exacerbation rates are increased among female, black, Hispanic, and younger adult patients: results from the US CHRONICLE study. J Asthma. (2022) 59:2495–508. doi: 10.1080/02770903.2021.2018701, PMID: 35000529

[6] Barnes PJ. New concepts in the pathogenesis of bronchial hyperresponsiveness and asthma. J Allergy Clin Immunol. (1989) 83:1013–26. doi: 10.1016/0091-6749(89)90441-7, PMID: 2659643

[7] Brannan JD Lougheed MD. Airway hyperresponsiveness in asthma: mechanisms, clinical significance, and treatment. Front Physiol. (2012) 3:460. doi: 10.3389/fphys.2012.00460, PMID: 23233839 PMC3517969

[8] Cockcroft D. Environmental causes of asthma. Semin Respir Crit Care Med. (2018) 39:12–8. doi: 10.1055/s-0037-1606219, PMID: 29427981

[9] Coates AL Wanger J Cockcroft DW Culver BH the Bronchoprovocation Testing Task Force Carlsen KH. ERS technical standard on bronchial challenge testing: general considerations and performance of methacholine challenge tests. Eur Respir J. (2017) 49:1601526. doi: 10.1183/13993003.01526-2016, PMID: 28461290

[10] Kraemer R Smith HJ Sigrist T Giger G Keller R Frey M. Diagnostic accuracy of methacholine challenge tests assessing airway hyperreactivity in asthmatic patients - a multifunctional approach. Respir Res. (2016) 17:154. doi: 10.1186/s12931-016-0470-0, PMID: 27855687 PMC5114725

[11] Ortega H Bharmal N Khatri S. Primary care referral patterns for patients with asthma: analysis of real-world data. J Asthma. (2022) 60:609–15. doi: 10.1080/02770903.2022.2082308, PMID: 35620831

[12] Bateman ED Hurd SS Barnes PJ Bousquet J Drazen JM FitzGerald M. Global strategy for asthma management and prevention: GINA executive summary. Eur Respir J. (2008) 31:143–78. doi: 10.1183/09031936.00138707, PMID: 18166595

[13] Holguin F Cardet JC Chung KF Diver S Ferreira DS Fitzpatrick A. Management of severe asthma: a European Respiratory Society/American Thoracic Society guideline. Eur Respir J. (2020) 55:1900588. doi: 10.1183/13993003.00588-2019 31558662

[14] Kim Y Lee H Chung SJ Yeo Y Park TS Park DW. The usefulness of FEF25-75 in predicting airway hyperresponsiveness to mannitol. J Asthma Allergy. (2021) 14:1267–75. doi: 10.2147/JAA.S318502, PMID: 34737579 PMC8560169

[15] Ciprandi G Cirillo I. The pragmatic role of FEF25-75 in asymptomatic subjects, allergic rhinitis, asthma, and in military setting. Expert Rev Respir Med. (2019) 13:1147–51. doi: 10.1080/17476348.2019.1674649, PMID: 31564180

[16] Siroux V Boudier A Dolgopoloff M Chanoine S Bousquet J Gormand F. Forced midexpiratory flow between 25 and 75% of forced vital capacity is associated with long-term persistence of asthma and poor asthma outcomes. J Allergy Clin Immunol. (2016) 137:1709–1716.e6. doi: 10.1016/j.jaci.2015.10.029, PMID: 26688518

[17] Xue Y Bao W Zhou Y Fu Q Hao H Han L. Small-airway dysfunction is involved in the pathogenesis of asthma: evidence from two mouse models. J Asthma Allergy. (2021) 14:883–96. doi: 10.2147/JAA.S312361, PMID: 34285515 PMC8286250

[18] McNulty W Usmani OS. Techniques of assessing small airways dysfunction. Eur Clin Respir J. (2014) 1:25898. doi: 10.3402/ecrj.v1.25898, PMID: 26557240 PMC4629724

[19] Postma DS Brightling C Baldi S van den Berge M Fabbri LM Gagnatelli A. Exploring the relevance and extent of small airways dysfunction in asthma (ATLANTIS): baseline data from a prospective cohort study. Lancet Respir Med. (2019) 7:402–16. doi: 10.1016/S2213-2600(19)30049-9, PMID: 30876830

[20] Usmani OS Singh D Spinola M Bizzi A Barnes PJ. The prevalence of small airways disease in adult asthma: a systematic literature review. Respir Med. (2016) 116:19–27. doi: 10.1016/j.rmed.2016.05.006, PMID: 27296816

[21] Kuwano K Bosken CH Paré PD Bai TR Wiggs BR Hogg JC. Small airways dimensions in asthma and in chronic obstructive pulmonary disease. Am Rev Respir Dis. (1993) 148:1220–5. doi: 10.1164/ajrccm/148.5.1220 8239157

[22] Cosio M Ghezzo H Hogg JC Corbin R Loveland M Dosman J. The relations between structural changes in small airways and pulmonary-function tests. N Engl J Med. (1978) 298:1277–81. doi: 10.1056/NEJM197806082982303, PMID: 651978

[23] Farah CS King GG Brown NJ Downie SR Kermode JA Hardaker KM. The role of the small airways in the clinical expression of asthma in adults. J Allergy Clin Immunol. (2012) 129:381–387.e1. doi: 10.1016/j.jaci.2011.11.017, PMID: 22188824

[24] Kjellberg S Houltz BK Zetterström O Robinson PD Gustafsson PM. Clinical characteristics of adult asthma associated with small airway dysfunction. Respir Med. (2016) 117:92–102. doi: 10.1016/j.rmed.2016.05.028, PMID: 27492518

[25] Bao W Zhang X Yin J Han L Huang Z Bao L. Small-airway function variables in spirometry, fractional exhaled nitric oxide, and circulating eosinophils predicted airway hyperresponsiveness in patients with mild asthma. J Asthma Allergy. (2021) 14:415–26. doi: 10.2147/JAA.S295345, PMID: 33907426 PMC8071078

[26] Yi L Zhao Y Guo Z Li Q Zhang G Tian X. The role of small airway function parameters in preschool asthmatic children. BMC Pulm Med. (2023) 23:219. doi: 10.1186/s12890-023-02515-3, PMID: 37340433 PMC10283187

[27] Malerba M Radaeli A Olivini A Damiani G Ragnoli B Sorbello V. Association of FEF25-75% impairment with bronchial hyperresponsiveness and airway inflammation in subjects with asthma-like symptoms. Respiration. (2016) 91:206–14. doi: 10.1159/000443797, PMID: 26855322

[28] Sposato B Scalese M Migliorini MG Di Tomassi M Scala R. Small airway impairment and bronchial hyperresponsiveness in asthma onset. Allergy, Asthma Immunol Res. (2014) 6:242–51. doi: 10.4168/aair.2014.6.3.242, PMID: 24843800 PMC4021243

[29] Peled M Ovadya D Cohn J Seluk L Pullerits T Segel MJ. Baseline spirometry parameters as predictors of airway hyperreactivity in adults with suspected asthma. BMC Pulm Med. (2021) 21:153. doi: 10.1186/s12890-021-01506-6, PMID: 33957916 PMC8101108

[30] Matsunaga K Kanda M Hayata A Yanagisawa S Ichikawa T Akamatsu K. Peak expiratory flow variability adjusted by forced expiratory volume in one second is a good index for airway responsiveness in asthmatics. Intern Med. (2008) 47:1107–12. doi: 10.2169/internalmedicine.47.0855, PMID: 18552467

[31] Yang Y Kimura H Yokota I Makita H Takimoto-Sato M Matsumoto-Sasaki M. Applicable predictive factors extracted from peak flow trajectory for the prediction of asthma exacerbation. Ann Allergy Asthma Immunol. (2024) 132:469–76. doi: 10.1016/j.anai.2023.11.015, PMID: 38006971

[32] Chen X Han P Kong Y Shen K. The relationship between changes in peak expiratory flow and asthma exacerbations in asthmatic children. BMC Pediatr. (2024) 24:284. doi: 10.1186/s12887-024-04754-7, PMID: 38678177 PMC11055252

[33] Douma W. R. Kerstjens H. A. Roos C. M. Koeter G. H. Postma D. S. Changes in peak expiratory flow indices as a proxy for changes in bronchial hyperresponsiveness. Dutch Chronic Non-Specific Lung Disease study group. Eur Respir J (2000) 16:220–225. doi: 10.1034/j.1399-3003.2000.16b07.x 10968495

[34] Ahmed A Brown A Pollack Y Vazhappilly J Perry C Thomas ER. Relationship between FEV1/FVC and age in children with asthma. Pediatr Pulmonol. (2024) 59:1402–9. doi: 10.1002/ppul.26927 38426807

[35] Mingotti C Sarinho J Stanigher K Silva J Roquette E Marchi E. Evaluating the FEV1/FVC ratio in the lower range of normality as a marker of worse clinical outcomes in asthmatic subjects without airway obstruction. Respir Med. (2020) 162:105880. doi: 10.1016/j.rmed.2020.105880, PMID: 32056671

