# Supplementary material for: Innovative machine learning-based prediction of early airway hyperresponsiveness using baseline pulmonary function parameters
Source: Front Med (Lausanne). 2025 Aug 4;12:1611683. doi: 10.3389/fmed.2025.1611683 (PMC12358348; doi:10.3389/fmed.2025.1611683)
Supplement: Supplementary file 1 [file Data_Sheet_1.pdf]

Supplementary Materials

Innovative machine learning-based prediction of early airway hyperresponsiveness using baseline pulmonary function parameters

Hua Yang[1,#,*], Xingru Zhao[2,#], Zhuochang Chen[2], Lihong Yang[1], Guihua Zhao[1], Chenxiao Xu[1], Jinyi Xu[1,*]

[1]Department of Cardiopulmonary Function, Henan Provincial People's Hospital, Zhengzhou University People's Hospital, Zhengzhou, Henan, 450003, China

[2]Department of Respiratory and Critical Care Medicine, Zhengzhou University People's Hospital, Henan Provincial People's Hospital, Zhengzhou, Henan, 450003, China

[*]Corresponding author. Emails: 18370960996@163.com & xjyecg@zzu.edu.cn.

[#]Hua Yang and Xingru Zhao contributed equally

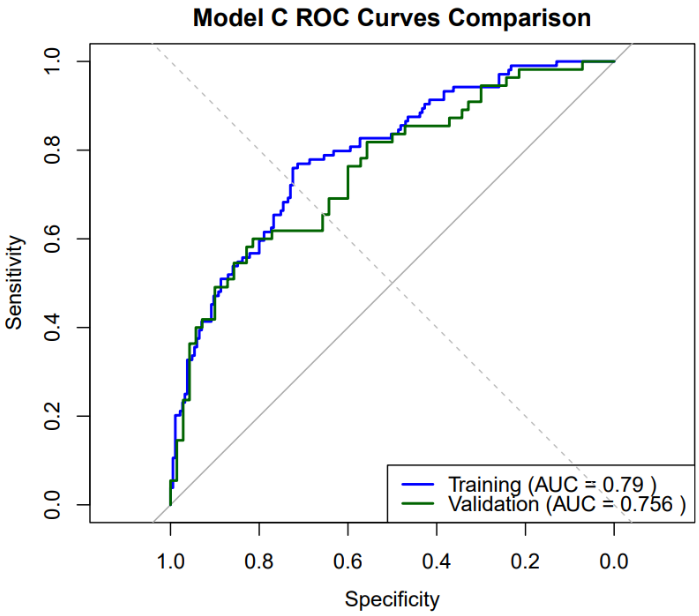

Figure S1. Application of DeLong's test to evaluate the statistical significance of AUC differences between the training and validation cohorts
